# Comparing measures of centrality in bipartite patient-prescriber networks: A study of drug seeking for opioid analgesics

**DOI:** 10.1371/journal.pone.0273569

**Published:** 2022-08-30

**Authors:** Kai-Cheng Yang, Brian Aronson, Meltem Odabas, Yong-Yeol Ahn, Brea L. Perry

**Affiliations:** 1 Luddy School of Informatics, Computing, and Engineering, Indiana University, Bloomington, IN, United States of America; 2 Department of Sociology, Indiana University, Bloomington, IN, United States of America; 3 Network Science Institute, Indiana University, Bloomington, IN, United States of America; RIKEN, JAPAN

## Abstract

Visiting multiple prescribers is a common method for obtaining prescription opioids for nonmedical use and has played an important role in fueling the United States opioid epidemic, leading to increased drug use disorder and overdose. Recent studies show that centrality of the bipartite network formed by prescription ties between patients and prescribers of opioids is a promising indicator for drug seeking. However, node prominence in bipartite networks is typically estimated with methods that do not fully account for the two-mode topology of the underlying network. Although several algorithms have been proposed recently to address this challenge, it is unclear how these algorithms perform on real-world networks. Here, we compare their performance in the context of identifying opioid drug seeking behaviors by applying them to massive bipartite networks of patients and providers extracted from insurance claims data. We find that two variants of bipartite centrality are significantly better predictors of subsequent opioid overdose than traditional centrality estimates. Moreover, we show that incorporating non-network attributes such as the potency of the opioid prescriptions into the measures can further improve their performance. These findings can be reproduced on different datasets. Our results demonstrate the potential of bipartiteness-aware indices for identifying patterns of high-risk behavior.

## Prescription opioid-seeking for nonmedical use

Visiting multiple health care providers is a widespread method for obtaining opioids for nonmedical use, and played an important role in fueling the United States opioid epidemic [1,2]. From 1999 to 2018, age-adjusted rates of mortality by opioid overdose increased by approximately 240%, from 6.1 per 100,000 in 1999 to 20.7 per 100,000 in 2018 [3]. For many of these years, increases in opioid overdose mortality rates mapped closely with increasing rates of opioid prescriptions and their nonmedical use [4,5]. Studies estimate that as many as 25% of individuals who die from opioid overdose—be it from prescription or illicit sources—had multiple simultaneous opioid prescriptions from different providers [2,6,7]. Given that users of nonmedical opioids tend to be geographically clustered [8,9] and tend to obtain opioids from relatively narrow sets of sympathetic, lax, or complicit opioid providers [10–12], it is believed that patterns of nonmedical opioid use correspond closely to patterns of deliberate prescription drug-seeking through patient social networks [13]. In other words, it is likely that users of nonmedical opioids leverage their social networks to identify and seek prescriptions from a small subset of opioid providers.

Although visiting health care providers to obtain opioids for nonmedical use is a critical predictor of opioid overdose, identifying this behavior among patients is a difficult task [13–16]. One common measurement tracks whether a patient had a multiple provider episode (MPE), defined by obtaining opioid prescriptions from multiple doctors simultaneously (commonly four) over a short period of time (commonly 90 days) [14,15]. However, classifying prescription drug-seeking using MPE results in estimates with high specificity but low sensitivity: very few patients obtain opioid prescriptions from multiple providers over a short period of time [17]. Yet, lower thresholds for measuring opioid-seeking through MPEs are equally problematic, as many patients who obtain opioid prescriptions from two or three doctors during a short time period often do so for legitimate (and often urgent) medical reasons.

A second common measurement for prescription opioid-seeking is based on the potency, or the total morphine milligram equivalent (MME), of all opioids prescribed to a particular patient during a particular interval of time [18]. However, MME does not always track closely with the true abusability of a patient’s prescriptions. Many opioids have high MME but have relatively low abuse rates due to their distinct pharmacological properties. For example, buprenorphine has high MME, but its slow absorption rate and its tendency to block certain opioid receptors in the brain substantially reduce its use among nonmedical opioid seekers. In fact, these properties make buprenorphine an effective medication for opioid use disorder [19]. In addition, many individuals who receive a large total MME of opioid prescriptions obtain them from only a single doctor, and many providers of high MME drugs are specialists (e.g., oncologists) that are unlikely to be identified and sought out by people that seek opioids for nonmedical use.

Because prescription drug-seeking is a *relational* behavior, several studies have proposed using social network analysis to identify it [12,17,20–22]. Patients who seek nonmedical prescription drugs typically visit providers who are disproportionately willing to prescribe opioids in an increasingly regulated environment. The providers may be sympathetic to pain patients who are longtime users of opioids and have developed a high tolerance. More problematic are providers who are unaware of the risks of opioid addiction, have poor training in identifying appropriate analgesic use, are easily exploited by fraudulent patients, or because the providers are directly engaged in illegal drug diversion [23,24]. For this reason, patients seeking prescriptions for nonmedical use are believed to visit these same sets of providers and to recommend such providers to each other [8,21,22]. In aggregate, shared preferences for providers and the tendency to find providers through patient referral networks should result in clustering of patients engaging in drug-seeking around the same sets of opioid providers. This would result in patients seeking opioids for nonmedical use holding prominent structural positions within patient-provider opioid prescription networks [23]. Based on this observation, two studies found that network centrality in a patient-provider opioid prescription network is strongly associated with other common measures of nonmedical prescription opioid use and that centrality is significantly more predictive of future opioid overdoses than are most traditional measures [17,21].

## Network centrality

Centrality is a key analytical tool in social network analysis, capturing important information about an individual’s prominence or role within a given network [25,26]. For example, a scientist’s centrality in a scientist-paper network captures the scientist’s degree of interdisciplinary bridging and opinion leadership [27,28]; a patient’s centrality in a patient-provider prescription network may reflect the extent to which the patient is deliberately searching for sympathetic, lax, or complicit opioid providers [21,23]. Both scientist-paper and patient-provider networks are examples of bipartite (or two-mode) networks, where social ties only occur across but not within two distinct sets (or modes) of actors [25,29]. In the case of scientist-paper networks, scientists are only connected through their shared publications; in patient-provider prescription networks, patients are only affiliated to each other through their shared providers.

Despite substantial research interest in measuring node prominence, many algorithms, such as PageRank [30] or eigenvector centrality, are designed primarily for unipartite (one-mode) networks. In these cases, social ties are assumed to be unconstrained and thus possible between every pair of nodes [31]. These centrality algorithms cannot take into account the fact that ties within each mode of a bipartite social network are never present; therefore, when applied to bipartite social networks, these centrality algorithms can provide misleading results [32,33]. Indeed, the biases induced by applying eigenvector-based centrality algorithms to bipartite social networks have been a source of concern in social network research for decades [25,31,34,35]. A common workaround is one-mode projection, which reduces the bipartite network into a unipartite network consisting of only one mode of nodes, then calculates the centrality indices [21,35]. Connections are allowed between all node pairs in the projection, circumventing the challenge brought by the bipartite nature of the original network. Yet, the projection might cause information loss and distort the network topology [36], also leading to misleading results.

To address these issues, centrality indices specifically designed for bipartite networks, such as HITS, CoHITS, BGRM, and BiRank, have been developed [35,37–39]. They operate similarly to eigenvector centrality and PageRank in that they iteratively update node centrality estimates based on each node’s connectivity and walk distance to other prominent nodes in the social network; however, bipartite algorithms differ in that they explicitly consider bipartite structure and are likely to produce more accurate estimates of node prominence.

To confirm and compare the effectiveness of bipartiteness-aware node prominence index in identifying opioid drug seekers, the present paper studies four variants of bipartite centrality index, i.e., HITS [40], CoHITS [37], BGRM [39], and BiRank [38], in a real-world social network of patient-provider relationships. We also project the bipartite patient-provider network into a unipartite patient-patient network and apply PageRank to produce a baseline [17,21]. Lacking the ground truth of drug seeking behavior, we use opioid overdose diagnosis as the outcome and evaluate how strongly each index is associated with the outcome for patients receiving opioid prescriptions. We further take advantage of the flexibility of bipartite centrality indices and incorporate attribute of patient-provider ties into the estimates. We illustrate how doing so can improve our ability to capture specific aspects of prescription drug-seeking that centrality on the unweighted network cannot.

## Centrality in bipartite networks

In this section we introduce the bipartite centrality indices tested in this paper. We start with the original PageRank index then describe how it can be expanded to bipartite networks. Finally, we describe how bipartite centrality indices can incorporate non-network information as edge weights to better capture the characteristics of the underlying social processes.

### PageRank centrality index on one mode network projection

PageRank is a specialized variant of eigenvector centrality that measures the extent to which a node is connected to other prominent nodes in a social network [30,41]. Essentially, PageRank estimates the stationary probability distribution of random walkers over all nodes [33]. Nodes that can be easily reached by random walkers, e.g., by having many incoming edges, are assigned larger PageRank scores.

The algorithm for PageRank is formalized as follows:

$$R(i)=\alpha \sum_{j\in M(i)}\frac{R(j)}{k_{j}^{out}}+(1-\alpha )R^{0(i)},$$ (1)

*R*(*i*) represents the PageRank score of node *i*, α indicates a damping factor assigned to the random walk (typically set to 0.85), *M*(*i*) represents the set of nodes that point to node *i*, $k_{j}^{out}$ indicates the out-degree of node *j*, and *R*^0(*i*)^ is typically set to $\frac{1}{N}$ where *N* represents the size of the network. The algorithm first divides node ranks by the degree of each node $\frac{R(j)}{k_{j}^{out}}$ to normalize the calculation, which is its key difference from eigenvector centrality. In matrix notation, if **R** represents a vector of PageRank values for all the nodes and *S* represents the normalized transition matrix, PageRank can be expressed as:

$$R=\alpha SR+(1-\alpha )R^{0}$$ (2)

Although PageRank and other variants of eigenvector centrality were designed for unipartite graphs, these algorithms are frequently applied to studies of bipartite networks. However, when they are applied, the original network is usually converted to a unipartite network. The most common methods for converting bipartite networks to unipartite networks are (1) to take the cross product of the original network such that the resulting network represents transitive ties between nodes on a single mode of the original network [29,32,42,43], (2) to treat the network as if ties were possible within modes by adding empty rows for each node that was only represented in the columns of the original network and vice-versa [25,31], or (3) to dual project the network by estimating ranks on each mode of the network separately before combining the network back into its original form [34,44–46]. These conversion methods can interact with some centrality algorithms differently than others, but for PageRank and other variations of eigenvector centrality, each method returns nearly identical node ranks [31]. At the same time, such conversion methods always result in some degree of network distortion that can bias centrality estimates or even give rise to wholly spurious network structures [42,47].

Within a patient-provider network, we expect PageRank to measure a patient’s network centrality and likely prescription drug-seeking behavior to a large extent, albeit somewhat imprecisely. PageRank is likely to assign high centrality estimates to patients connected to frequent opioid providers and to patients who see the same sets of doctors as others who may be seeking drugs for nonmedical use. However, PageRank on the one mode projection of a patient-provider network will treat all transitive ties as having the same structural importance. This means that a patient with only one provider tie could have the same degree in the projected network as a patient who has ties to multiple weakly-connected doctors. Since PageRank is highly influenced by a node’s degree, this feature of the one-mode network projection is likely to significantly bias how well PageRank captures true prescription drug-seeking behavior. We expect some patients with connections to one or two well-connected providers to have high PageRank estimates, while patients with connections to larger number of poorly connected providers may have low PageRank estimates. Both outcomes are exactly the opposite of how we would typically want to define and operationalize patients who visit multiple providers to obtain opioids for nonmedical use. In addition, the one mode projection may distort the estimated patient behavior in other subtle but not necessarily predictable ways.

### Bipartite centrality indices

Bipartite centrality indices are a direct extension of the PageRank index and follow the intuition that a vertex should have higher centrality if it is connected to other vertices with high centrality. Like PageRank, bipartite centrality algorithms update centrality estimates for nodes iteratively based on each node’s connection to others and based on the most recent rank estimates of each node’s alters. However, bipartite algorithms differ from PageRank in their ability to estimate centrality directly on a bipartite network simultaneously for both modes of the network. Bipartite centrality algorithms respect the absence of ties between nodes within the same mode of the network, and the necessity for centrality to only compare nodes within the same mode of a network. In every iteration, bipartite centrality algorithms update centrality estimates for every node in the network based on their connections to other nodes in the network, their centrality estimates in the previous stage of the algorithm, and by their connections’ centrality estimates in each stage of the algorithm. Put together, bipartite centrality algorithms propagate the centrality estimates back and forth across each mode of the network throughout each iteration of the algorithm.

The bipartite centrality algorithms tested in this study are HITS, CoHITS, BGRM, and BiRank [37–40]. These algorithms were developed separately and for distinct purposes, but many of their underlying similarities have been identified and systematized in recent work by He et al. [38] The algorithm underlying all bipartite centrality measures is formalized as follows:

$$p(j)=\alpha \sum_{i=1}^{D}\frac{w_{ij}}{S_{p(i)}^{*}}d(i)+(1-\alpha )p^{0}(j),$$

$$d(i)=\beta \sum_{j=1}^{P}\frac{w_{ij}}{S_{d(j)}^{*}}p(j)+(1-\beta )d^{0}(i)$$ (3)

Here *p*(*j*) and *d*(*i*) represent the bipartite centrality of patient *j* and provider *i* separately, α and β represent the damping factors for the random walk (typically set to 0.85), D and P represent the number of providers and patients in the network, *w*_*ij*_ indicates the element of the adjacency matrix *W*^*D*×*P*^ at coordinate (*i*,*j*). $S_{p(i)}^{*}$ and $S_{d(j)}^{*}$ represent normalizers that differ across each bipartite centrality algorithm and are outlined further below. In matrix notation, if **p** and **d** represent a vector of centrality estimates of every node in each mode of the network and *S*_*p*_ and *S*_*d*_ represent transition matrices of the network across iterations, the bipartite centrality algorithm can be expressed as:

$$p=\alpha S_{p}d+(1-\alpha )p^{0},$$

$$d=\beta S_{d}p+(1-\beta )d^{0}$$ (4)

Variations of the bipartite centrality algorithm differ primarily in how they normalize the network prior to iteration. Kleinberg’s HITS (Hyperlink-Induced Topic Search) was originally designed for estimating node prominence in one mode networks by iteratively ranking nodes according to their role as an “authority” in the network and their role as a “hub” in the network [40]. Nodes with high authority scores are defined by having a high indegree from high-ranking hubs; and nodes with high hub scores are defined by having a high outdegree to nodes with high authority. The version of HITS tested here was slightly expanded by He et al. to interface with bipartite networks [38], but is otherwise identical to the algorithm proposed by Kleinberg. HITS differs from every other bipartite centrality index in that it does not normalize the input adjacency matrix prior to initializing its iteration process, analogously to the eigenvector centrality. To ensure convergence, HITS normalizes centrality estimates after every iteration by dividing each node’s rank value by the sum of all rank values on that mode. The design of HITS fully respects the bipartite network topology. However, HITS is known for its tendency to produce unintuitive centrality estimates, even in comparison to PageRank [48]. There are a variety of network features that can lead to this problem, but HITS’ most central weakness is its sensitivity to outliers. Nodes with high degrees can have an extreme and often unexpected influence on how HITS ranks all other nodes in the network. A similar phenomenon is the localization of Eigenvector centrality onto a small number of hub nodes [49].

CoHITS [37] is named for its ability to better-incorporate content information into its algorithm than HITS (the “Co” in CoHITS). Unlike HITS, CoHITS normalizes the input network prior to iterations to avoid divergence. In particular, CoHITS normalizes the adjacency matrix by the outdegree of the source nodes, simulating random walks on the bipartite networks. In other words, CoHITS estimates reflect the probability distribution of finding the random walkers on the nodes. Considering this interpretation, CoHITS is very similar to PageRank when applied directly to bipartite networks. The major difference is that CoHITS ranks the nodes from two modes separately while PageRank is not aware of the bipartite nature of the network. A possible weakness of CoHITS is that it only normalizes the network on the source nodes; nodes with high indegrees might still exert an undue influence on the estimates [38].

BGRM (Bipartite Graph Reinforcement Model) was developed for automating web image annotation [39]. Like CoHITS, BGRM also normalizes the adjacency matrix to avoid divergence. The difference is that BGRM adopts a symmetrical weighting scheme, whereby an edge is normalized by the degree of both ends of the vertex simultaneously. This design reduces the impact of high indegree nodes but might introduce other biases. For example, BGRM may assign, in the case of patient-provider network, overly low centrality estimates to high degree patients that are connected to high degree providers and overly high centrality estimates to low degree patients that are connected to low degree providers.

BiRank is the newest bipartite centrality index and was developed to improve upon the theoretical advantages of BGRM’s symmetric normalization scheme [38]. BiRank divides each edge by the square root degree of the source node and the square root degree of the target node prior to iteration. This means that BiRank exerts a significantly lower degree of normalization on the inputted network than BGRM, and in theory, should be less prone to outliers on either mode of the network than CoHITS.

A summary of the differences among the bipartite centrality algorithms’ normalization schemes are formalized in Table 1.

**Table 1:** Summary of bipartite centrality algorithms’ normalization schemes.

Algorithm	Transition Matrix of Top Mode (*S*_*p*_)	Transition Matrix of Bottom Mode (*S*_*d*_)
HITS	*W* ^*T*^	*W*
CoHITS	$W^{T}K_{d}^{-1}$	$WK_{p}^{-1}$
BGRM	$K_{p}^{-1}W^{T}K_{d}^{-1}$	$K_{d}^{-1}WK_{p}^{-1}$
BiRank	$K_{p}^{-1/2}W^{T}K_{d}^{-1/2}$	$K_{d}^{-1/2}WK_{p}^{-1/2}$

*K*_*d*_ and *K*_*p*_ represent diagonal matrices with the generalized degrees (sum of edge weights) on the diagonal of each mode of the network. Specifically, (*K*_*d*_)_*ii*_ = ∑_*i*_*w*_*ij*_ and (*K*_*p*_)_*jj*_ = ∑_*i*_*w*_*ji*_.

### Edge weights in bipartite centrality indices

Some important properties of nodes are not captured in the network structure itself. Focusing just on the network structure, we might ignore important information about why some nodes are more central than others. In our case, the tendency for patients to seek providers who administer large quantities of potent opioids is not captured but may be critical for predicting outcomes like overdose. The bipartite centrality indices considered here provide an interesting solution; we can assign weights to provider-patient ties by a relevant edge attribute — the total morphine milligram equivalent (MME) of opioids prescribed to the patient. MME is a common measurement for estimating the potency of opioid drugs that is based on the equivalence of an opioid prescription with 1mg of orally taken morphine per day. MME typically refers to the potency of a single dosage of an opioid, but some scholars examine the total MME of all pills prescribed to a particular patient to estimate the potency and quantity of opioids prescribed, and occasionally, to track potential fraudulent behavior and nonmedical use [18]. By weighting each edge of the patient-provider network by the total MME of prescriptions administered by a provider to a particular patient, we improve our measurement of centrality by incorporating patient preferences for providers that administer large quantities of highly potent opioids. This strategy should be particularly effective for reducing the centrality estimates of patients who are connected to providers who only administer short term analgesic treatments, such as surgeons and emergency care physicians.

## Methods

### Data

Data are drawn from Optum’s de-identified Clinformatics® Data Mart Database, a national, commercial, and Medicare Advantage claims database that contains dated information about patient medical histories, office appointments, and prescriptions during the years 2007 to 2018. The sample does not include medical claims across all providers, but there are many regions where the medical claims database covers the plurality of patient-provider visits. We lose coverage of patients who switch medical plans over time; however, most patients have multiple years of data in the sample. Due to our theoretical interest in opioid prescription drug-seeking, we limit our sample to patients who have ever received an opioid prescription, identified via their national drug codes (NDCs). Patient-quarters (three-month intervals) comprise our unit of analysis. This study was approved by the Indiana University Institutional Review Board. Owing to the use of deidentified patient data, the need for informed consent was waived.

To ensure an optimal context for comparing different measures of patient behavior, we focus our analysis to 2009 quarter 2 through 2012 quarter 2. We choose this time period because these years had very high rates of prescription opioid abuse [5,50]. By 2012, many states implemented prescription drug monitoring programs (PDMPs) that curtailed rates of fraud and abuse and contributed to a rise in consumption of more easily accessible illicit opioid substances that are not prescribed by health professionals [51,52]. In other words, focusing on the years 2009 to 2012 reduces the degree to which use of illicit opioids (e.g., heroin, fentanyl) is likely to create noise in our predictions of opioid overdose via prescription drug-seeking.

**Fig 1:**
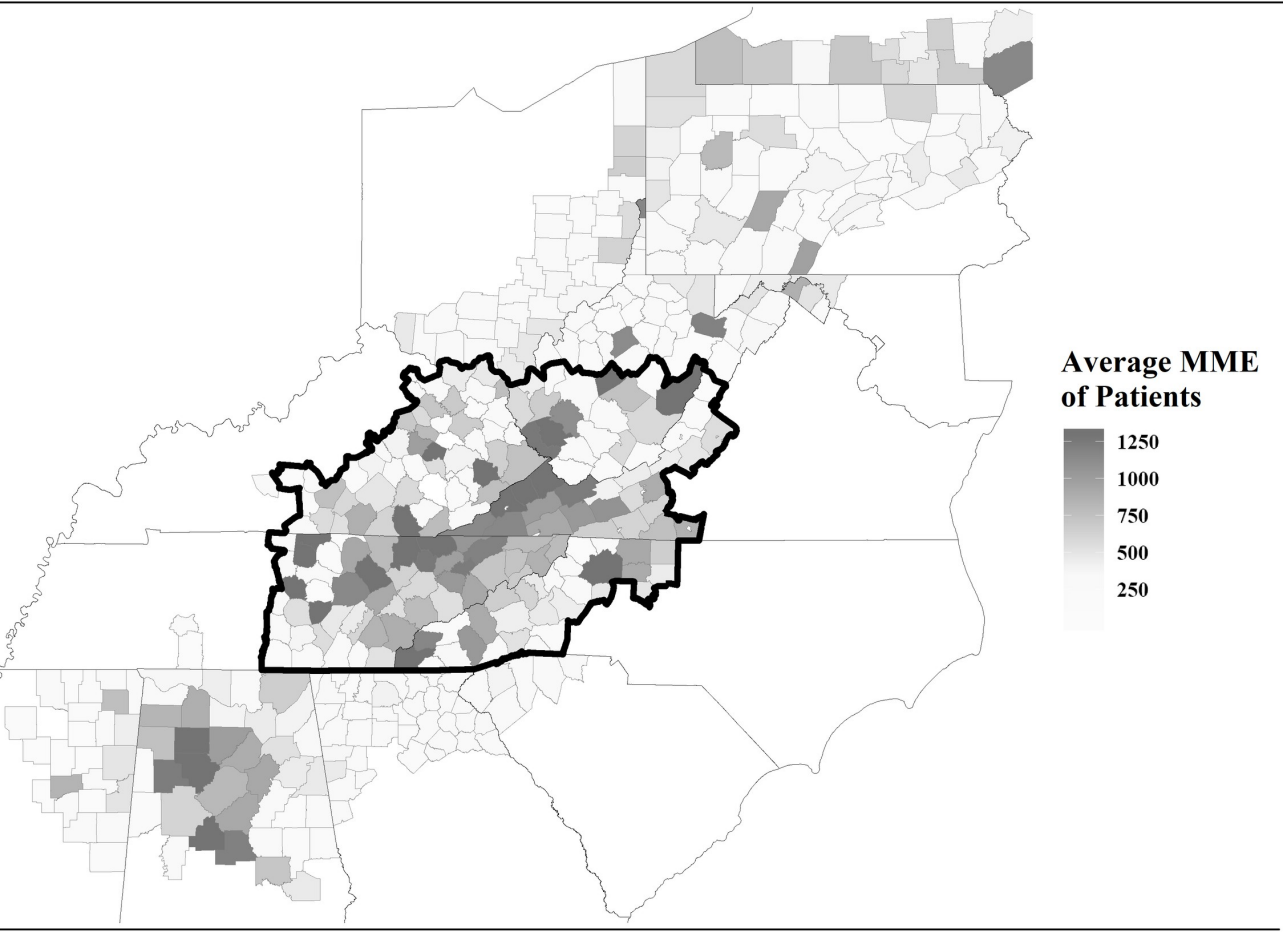
Analytic sample within Appalachia. Thick borders indicate sample boundaries; medium borders indicate state boundaries; thin borders indicate county boundaries. Different shades represent the average MME of patients for all counties in Appalachia. The estimates are based on the first quarter of 2011. The figure was generated using the TIGER/Line Shapefiles product provided by the U.S. Census Bureau [57].

We also focus our analytic sample on a narrow region in the U.S. Appalachian Mountain range. Appalachia is a well-known hotspot of opioid overdoses in the U.S. Moreover, narrowing analysis to a small geographic region reduces the complexity of our methodological approach. Specifically, physical proximity plays a large role in which providers a patient chooses to seek opioid prescriptions from. Estimating centrality across a network that spans a broad geographic region would likely induce network endogeneity that biases the degree to which centrality reflects true drug-seeking behavior. Although there are a variety of methods for accounting for network endogeneity [53–56], narrowing our analysis to a small geographic region is the most parsimonious option. Since we are primarily concerned with comparing different measures of centrality, we endeavor to keep our analytic approach as straightforward as possible. The geographic region of our analytic sample and corresponding rates of opioid overdose within Appalachia are illustrated in Fig 1. The boundary of the analytic region is composed of county lines across five states, including West Virginia, Virginia, North Carolina, Kentucky, and Tennessee. In total, our subsample within this region and time period includes 245,133 unique patients, 38,486 unique prescribers, and 1,920,554 patient-quarters.

### Network formation

We use separate intervals from the opioid prescription dataset to construct a longitudinal bipartite social network of patient-provider ties. Ties are defined by whether a patient received at least one opioid prescription from a provider during a given interval. Repeated ties are not treated as valued in the unweighted bipartite network, but we do give weight to repeated transitive ties in the one-mode network projection with PageRank centrality, and we indirectly give weight to repeated ties in our secondary analyses of the MME-weighted patient-provider network. Although our unit of analysis is patient-quarters, we construct the longitudinal bipartite social network with three-quarter (nine month) rolling windows of patient-provider prescriptions. We made this choice to better reflect the duration of true drug-seeking behavior. With one quarter intervals, we noticed that some patients exhibited periods of limited behavior directly preceded and followed by extreme periods of prescription drug-seeking. These short gaps do not have any clear negative association (or lagged negative association) with reduced rates of opioid overdose. We believe it is unlikely that such patients truly stopped seeking drugs during these short intervals. Rather, these patients either had short periods of difficulty in successfully attaining opioid prescriptions or short periods of reprieve in visiting providers due to their recent successes in attaining drugs either legally or illegally. However, to ensure that our analyses are not biased by our choice to construct the patient-provider network with three-quarter rolling windows of prescription data, we re-estimate all models with networks comprised of only a single quarter of prescription data and find that these yield substantively identical results to those constructed using three quarters.

### Statistical models

We compare centrality estimates of prescription drug-seeking behavior by examining their ability to predict subsequent opioid overdose. We estimate the degree to which each independent and control variable is associated with a patient’s conditional probability (hazard) of opioid overdose over time with Cox proportional hazard models [58], a type of event history model that are often used for studying the association between uncommon events with multiple predictors [59]. One key advantage of Cox models over traditional regression methods is their ability to prevent censorship bias. Many patients were dropped from the sample for reasons unrelated to opioid overdose (often because they switched employers and insurance providers), while some others died from opioid overdose and were censored due to their death. If we used logistic regression rather than Cox regression, censorship would cause bias in parameter estimates because the missing information is related to the dependent variable. In contrast, Cox models prevent censorship bias by removing censored observations from the risk set; censored individuals only contribute to hazard estimates during the years in which they were present. A second key advantage of Cox proportional hazard models is that they are semi-parametric. Traditional regression methods assume that data points lie across a particular distribution of event-times, be it normal, logistic, or Poisson, but Cox models make no such assumptions. This is critical for our analysis because we have no *a priori* hypothesis about the distribution of opioid overdose event times. Overdose events are rare and are unlikely to follow any typical probability distribution.

To compare the extent to which different measurements of centrality are representative of prescription drug-seeking behavior and predictive of opioid overdose, we estimate separate Cox proportional hazard models for each measurement of centrality. We manipulate each model specification only by the type of centrality measure included to identify patient behavior; we do not include all centrality measures at once because the measures are highly collinear. We compare the degree to which each model fits the data based on their resulting Akaike Information Criterion (AIC) [60]. AIC estimates a statistical model’s relative quality by using its maximum likelihood of each model while penalizing for each additional estimated parameter, such that models that over fit will have worse AIC than models with fewer parameters. A model with a substantially lower AIC value than others considered can be statistically shown to fit the data better than the other. For this reason, we use AIC to measure which centrality index best reflects true prescription drug-seeking behavior.

### Variables

The dependent variable across models is opioid overdose, defined by whether a patient was marked with a corresponding International Classification of Diseases 9^th^ edition code (ICD-9) [61]. Given that opioid overdose events are rare and the substances underlying opioid overdoses are often mixed and ambiguous, we do not attempt to distinguish patients who had overdosed on illicit or unspecified narcotic substances from those who had overdosed on prescribed opioids. To improve our ability to estimate the causal effect of patient behavior on opioid overdose and account for the tendency of prescription drug-seeking to precede opioid overdose, we also lag this dependent variable by one quarter. In total, our sample includes 390 overdose events.

The key independent variable across models is patient centrality. Centrality is estimated with HITS, CoHITS, BGRM, and BiRank via the BiRank statistical package [62]. We also apply PageRank on the one-mode network projection to provide a baseline. Although many other network centrality indices are available, these methods do not necessarily work well on large networks. For instance, eigenvector centrality is known to localize to a small set of nodes in large networks [49]. PageRank, on the other hand, has been proven robust and informative in the current context according to the literature [17,21]. We normalize all centrality measures across network components to keep centrality estimates at a consistent scale, and we take the natural log transformation of each centrality measure to account for their right-skewed distribution of centrality estimates. Finally, the transformed centrality measures are converted to *z*-scores. To ensure that the choice to take the natural log transformation of each centrality measure did not bias our estimates, we also estimated all models with centrality measures that were left untransformed and with centrality measures that were dichotomized above and below the 99^th^ percentile. Across all transformations of centrality, each model yielded substantively similar results.

We include a variety of independent variables to control for any spurious relationship between centrality and opioid overdose. We include age (in years) and sex (female) to reflect the slightly increased propensity for opioid overdose by people of middle age and male sex. We examine two individual-level network parameters that are likely associated with patient drug-seeking behavior, including patient degree (number of unique providers) and transitive ties (number of connections to other patients through one’s providers). These network parameters are likely to be associated with patient behavior, but neither of these parameters closely reflect a patient’s position within the entire structure of the patient-provider network. More specifically, neither network parameter reflects the tendency for patients to cluster around the same sets of lenient providers or to seek providers who tend to administer large quantities of potent opioids. Excluding these individual-level network parameters generated more statistically significant parameter estimates for network centrality; however, we include these parameters as an indirect test for whether network centrality provides any distinct contribution to predicting opioid overdose beyond traditional measures of drug-seeking behavior based on individual-level characteristics. Finally, we control for patients who are likely to receive more opioid prescriptions due to having a particular disease rather than due to nonmedical opioid use. These controls include whether a patient had hepatitis C (HEPC), human immunodeficiency virus (HIV), cancer, a psychiatric disorder, was receiving medication assisted treatments (MAT), or medication for opioid use disorder, or whether the patient was in palliative care.

## Results

### Visualization of the patient-provider network

**Fig 2:**
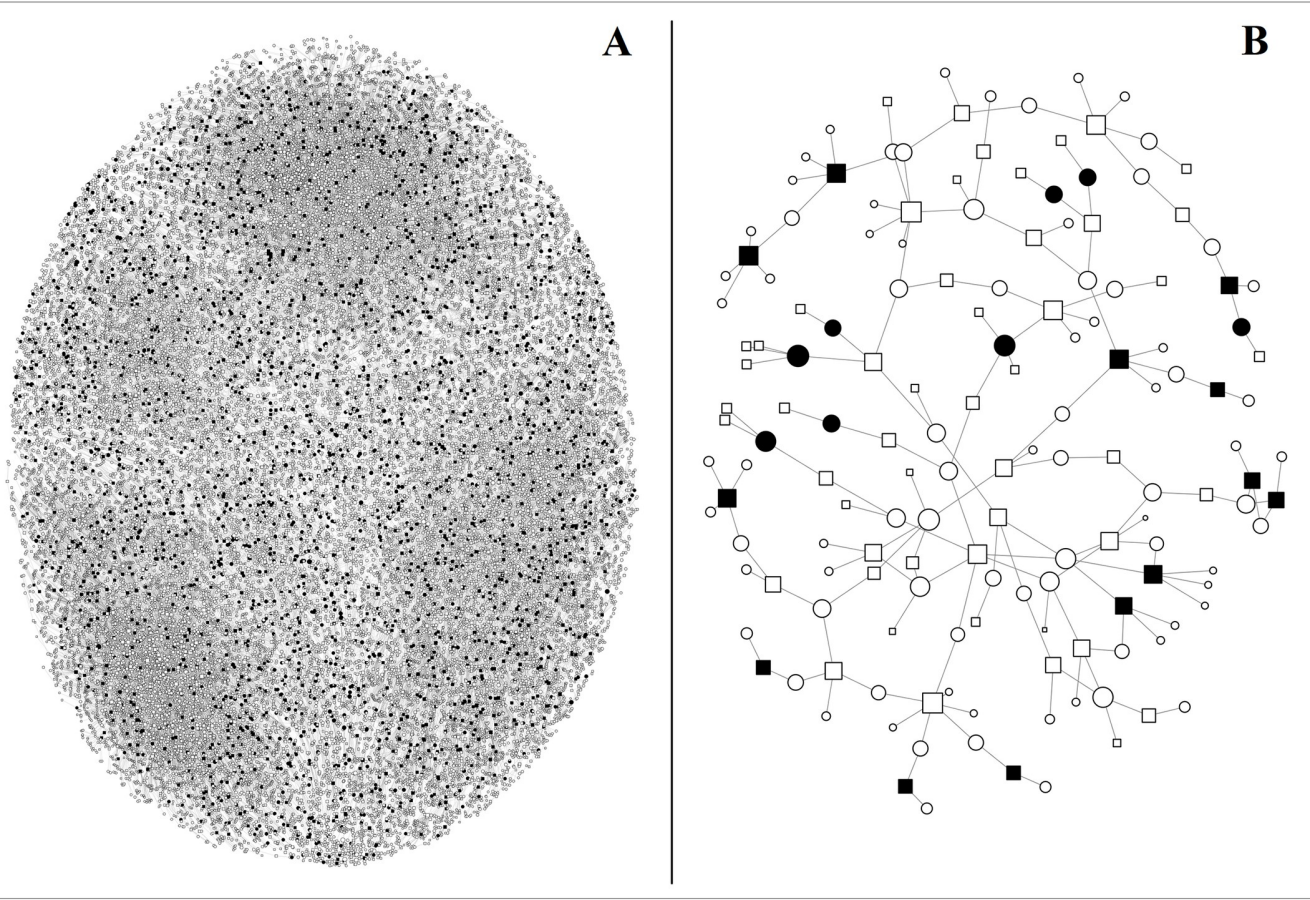
Sociogram of two largest network components. **A** indicates the largest network component; **B** indicates second largest network component. Networks are constructed from all opioid prescription ties from October 1, 2008 to June 30, 2009. Nodes are sized according to their BiRank centrality. Circular nodes indicate patients; square nodes indicate providers. To highlight the discrepancy between BiRank and PageRank estimates for the same nodes, the nodes are colored black if their BiRank value is two standard deviations above the mean BiRank value and if their PageRank value is below the mean PageRank value.

To orient readers to the analytic sample, we first illustrate the largest and second largest connected components of the patient-provider network during the first period of analysis (October 1, 2008 to June 30, 2009) in Fig 2A and 2B. The total number of patients sampled during the first period of analysis is 148,182, but during this period, only 41,698 patients received opioids from any doctor and are represented in the network. The largest component of the network comprises 33,024 patients or 79.2% of patients who received any opioids, 8,273 providers, and 49,738 edges, and the second largest component of the network comprises 91 patients or 0.2% of patients who received any opioids, 64 providers, and 164 edges. The high coverage of the largest connected component implies that patient-provider ties in this region of Appalachia represent a relatively coherent and well-connected network. Another notable pattern in Fig 2 is that no nodes appear to have extreme degrees. Although Fig 2A contains four somewhat denser clusters of nodes, there are many visible nodes and ties that lie between each cluster.

Nodes in each mode of the network in Fig 2 are scaled according to their BiRank centrality and shaped according to whether the node represents a patient (circle) or a doctor (square). To emphasize differences between BiRank and PageRank estimates, we color nodes black if their BiRank is two standard deviations above the mean BiRank and if their PageRank is below the mean PageRank. Nodes towards the center of Fig 2B and within the four clusters of Fig 2A tend to be grey, implying that PageRank and BiRank tend to assign similar ranks to well-connected nodes. Most black nodes appear towards the periphery of each network, implying that BiRank is more likely to assign above-mean centrality estimates for seemingly peripheral nodes than PageRank. However, a close examination of Fig 2B reveals that most patients with high BiRank and low PageRank are connected to multiple poorly connected providers in the global network. These patterns are consistent with our predictions: in comparison to BiRank, PageRank estimates relatively low centrality to high degree patients that are connected to low degree providers.

### Correlation between different centrality indices

**Fig 3:**
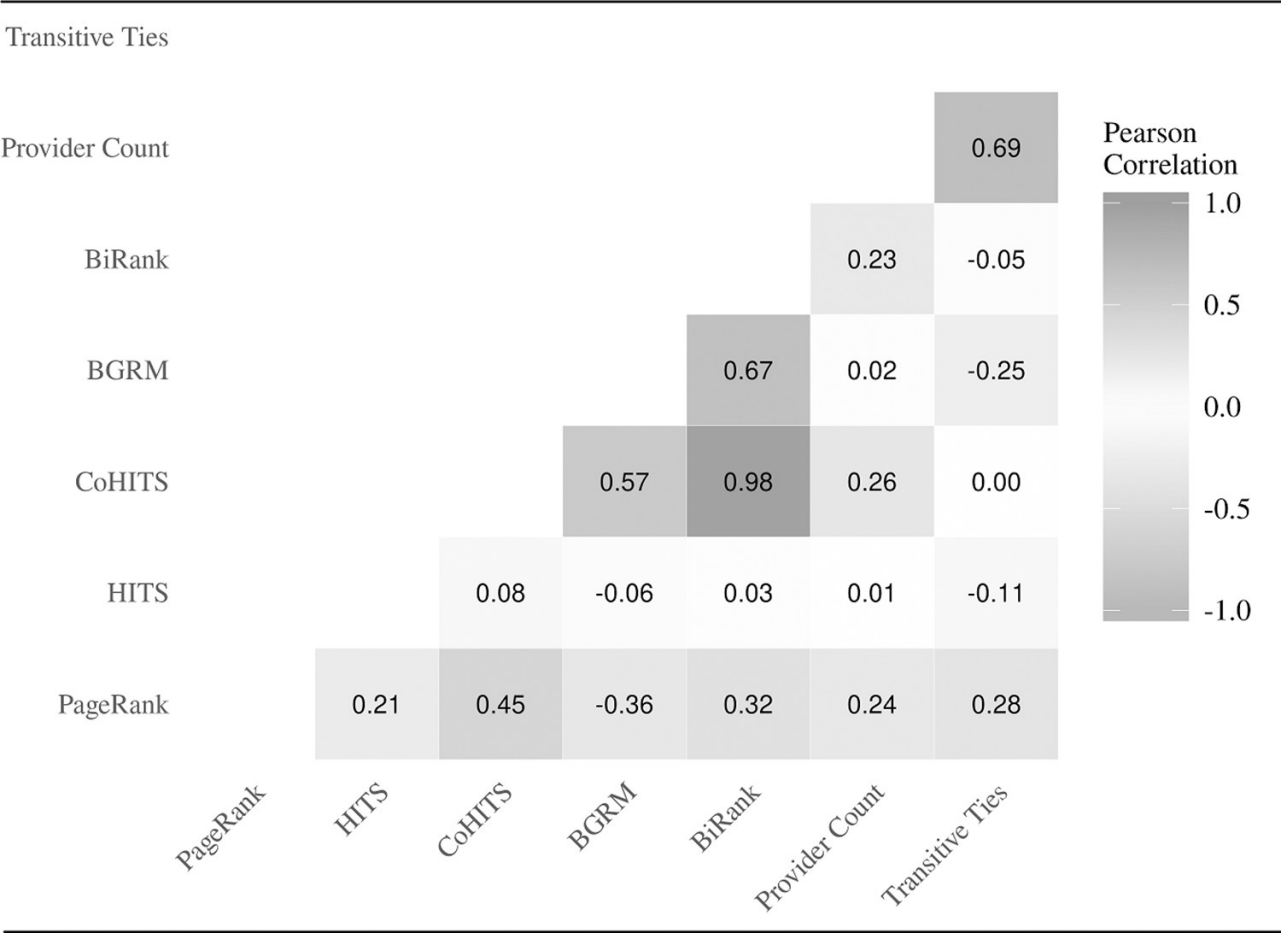
Correlation matrix of different centrality indices and key network parameters. The Pearson correlations are based on the full analytic sample (n = 1,920,554 patient-quarters). All variables are log-transformed. Spearman correlations yield qualitatively similar results.

We examine the relationship between different centrality indices and key network parameters by plotting their correlation matrix in Fig 3. Most centrality indices are moderately and positively correlated; however, HITS has a fairly low correlation with all other centrality methods and BGRM has a negative correlation with PageRank. BGRM’s negative correlation with PageRank is also reflected in its negative correlation with transitive ties, the latter of which is highly correlated with PageRank. On the other hand, most bipartite centrality indices have weak associations with transitive ties. Further exploration suggests that this weak association may result from a slight tendency towards negative degree assortativity (Pearson’s r = -0.03) in the provider-patient network; low degree patients tend to be connected to high-degree providers. Degree substantially contributes to bipartite centrality indices; therefore, it is understandable that the negative degree assortatitivity would induce a negative association between bipartite centrality and transitive ties. Another noteworthy correlation is the strong relationship between BiRank and CoHITS (Pearson’s r = 0.98). Even prior to log-transforming each variable, the correlation between BiRank and CoHITS is quite high (Pearson’s r = 0.96). This correlation is much stronger than the correlation between BiRank and BGRM, even though BiRank and BGRM both use symmetric normalization whereas CoHITS does not.

### Linear association between centrality indices and opioid overdose

**Fig 4:**
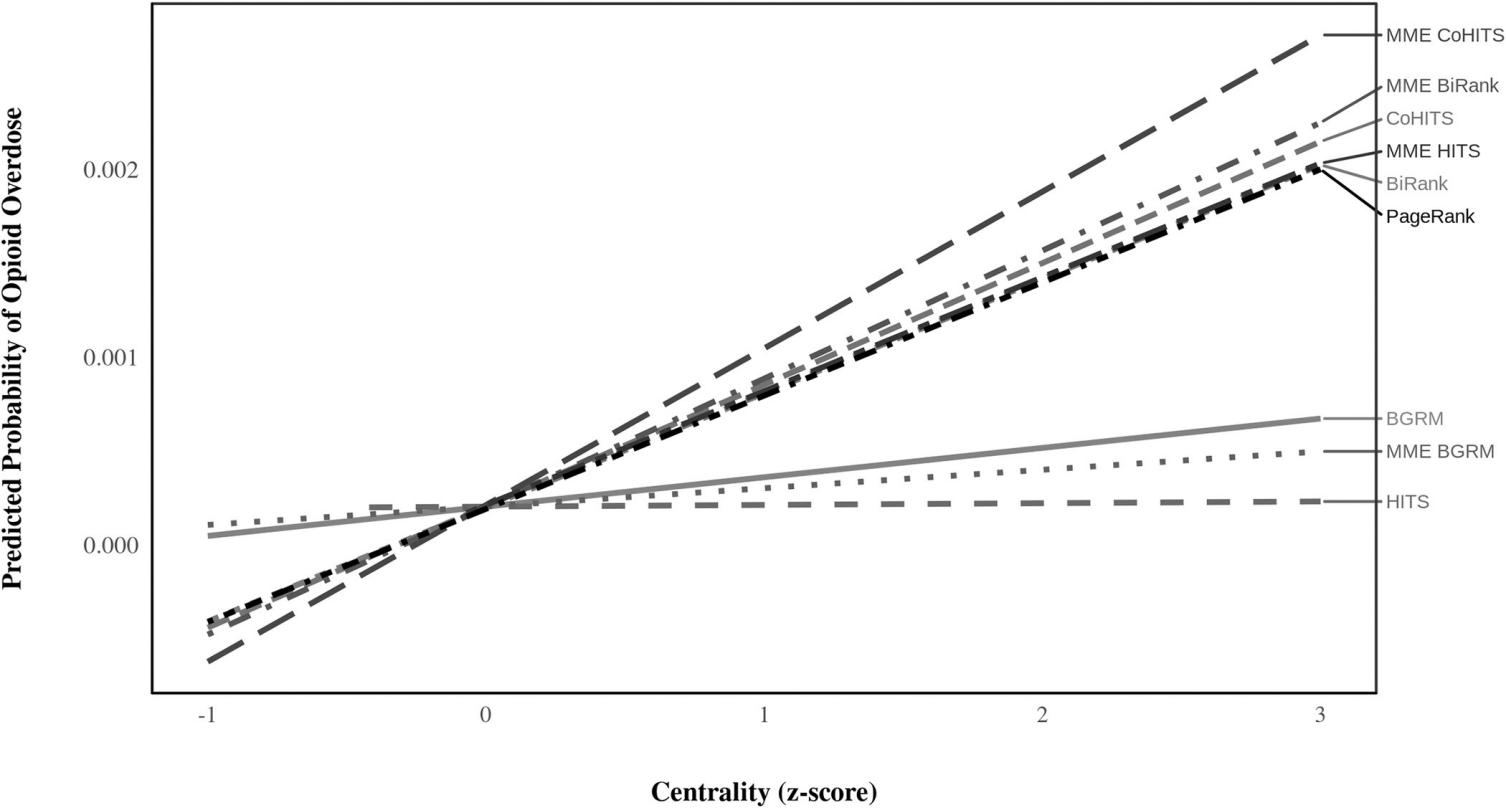
Linear association between network centrality indices and opioid. The centrality estimates are based on patient-provider network of opioid prescriptions. The slopes are based on the full analytic sample (n = 1,920,554 patient-quarters).

We illustrate the linear association between each weighted and unweighted centrality measure with opioid overdose in Fig 4. The association is derived from a simple linear regression: *Y* = *b*_0_+*b*_1_×*X*, where *Y* represents opioid overdose diagnoses, *b*_0_ is the intercept, *X* represents the *z*-scores of the centrality indices, and *b*_1_ measures the association. The results show how a one standard deviation change from the mean of a centrality score is associated with a patient’s predicted probability of having an opioid overdose diagnosis.

All centrality estimates are only modestly associated with subsequent opioid overdose, but this is expected given the infrequency of overdose events and the complicated set of conditions that cause such events. The centrality index that produces the greatest association with opioid overdose is CoHITS on the MME-weighted network, whereas HITS has the lowest association with opioid overdose. However, it is important to remember that these are raw associations that could be confounded by a variety of factors. The utility of any new benchmark for identifying drug-seeking behavior is largely based on the unique information that the algorithm can provide for predicting opioid overdose beyond that of traditional identifiers. Therefore, these patterns alone should not be interpreted as conclusive evidence that CoHITS on the MME-weighted network is the best predictor of opioid overdose. That said, the extremely limited association of opioid overdose with BGRM and HITS suggests that these algorithms may provide poor direct proxies of prescription drug-seeking behavior.

### Results of regression models

To account for confounding factors, we run multiple Cox proportional hazard models that estimate each patient’s hazard to overdose on opioids throughout the period of analysis. The results are shown in Table 2. Sample statistics about each variable are included in S1 Table. Across models, each parameter estimate illustrates the hazard ratio increase associated with a one standard deviation increase in that particular parameter. For example, Table 2 indicates that an individual with a logged BiRank score that is one standard deviation higher than another patient’s logged BiRank score has 1.28 times (or 28%) increased expected hazard of opioid overdose. We include a baseline model that only contains control variables. The other five models differ by their measures of network centrality. Parameter estimates for all centrality scores are positive, but are only statistically significant for CoHITS, BGRM, and BiRank.

**Table 2:** Cox proportional hazard models for opioid overdose.

	Baseline	PageRank	HITS	CoHITS	BGRM	BiRank
**Centrality**	-	1.064	1.040	1.217***	1.338***	1.279***
**Demographics**						
Age	0.920	0.930	0.924	0.957	0.936	0.963
Female	0.931	0.929	0.930	0.930	0.927	0.927
**Network Proxies**						
Degree (# Providers)	1.880***	1.844***	1.887***	1.576***	1.668***	1.540***
Transitive Ties^a^	1.338***	1.293***	1.319***	1.418***	1.606***	1.466***
**Related Disorders**						
HEPC	2.178	2.188	2.213*	1.974	1.947	1.985
HIV	2.677	2.678	2.713	2.696	2.510	2.657
Cancer	1.202	1.206	1.201	1.168	1.151	1.161
Psych Disorder	7.358***	7.322***	7.306***	7.130***	7.237***	7.107***
Palliative Care	4.904***	4.905***	4.995***	4.401***	4.510***	4.377***
MAT User^b^	2.488**	2.523**	2.439**	2.569**	2.407**	2.538**
AIC	9269	9269	9269	9248	9242	9244

* = p < 0.05

** = p < 0.01

*** = p < 0.001. Time-to-event is based on one quarter intervals between 2009 quarter 2 and 2012 quarter 2. There are n = 1,920,554 patient-quarters. The number of overdose events is 390. The parameters are standardized and reported as hazard ratios. a) Number of patient-patient ties through providers. b) Patient is receiving medication assisted therapy for opioid use disorder.

The other parameter estimates in Table 2 are mostly consistent with expectations. Age and gender are not significantly associated with opioid overdose, whereas degree and transitive ties are both significantly and positively associated with opioid overdose. We observe large increases in expected hazards for opioid overdose among individuals with psychological disorders, on palliative care, or on medication assisted treatments for opioid addiction. The associations of opioid overdose with HEPC, HIV, and cancer are not statistically significant, but the parameter estimate for each variable is positive and large. It is likely that these variables have positive parameter estimates but weak statistical significance due to the limited number of overdose cases in our sample.

The models’ AIC values in Table 2 indicate the degree to which the models fit the data. Compared with the baseline model, adding PageRank and HITS centrality measures to the regression models does not substantially alter the AIC value. In comparison to the model measuring centrality with PageRank, the BiRank model has an AIC value that is (9269–9244 =) 25 units lower. This indicates that the model with BiRank centrality index is significantly more likely to provide relevant unique information and improve model fit than the model with PageRank centrality (p = exp(-25/2); p < 0.001). Among the bipartite centrality indices, the model with HITS produces the highest AIC value although it is not substantially worse than the model with PageRank. On the other hand, models with CoHITS, BGRM, and BiRank have substantially lower AIC values. The differences among them are relatively small, for example, the difference in model fit between BGRM and CoHITS barely passes statistical significance at p = 0.05.

It is worth mentioning that BGRM has a much weaker linear association with opioid overdose than the other indices according to Fig 4. We run supplementary models without patient degree and transitive ties, and the model with BGRM is substantially worse than the models with BiRank or CoHITS. In other words, BGRM’s weak collinearity with degree increases the extent to which BGRM improves model fit relative to BiRank and CoHITS but limits its ability to directly proxy patient prescription drug-seeking behavior.

### Effectiveness of incorporating edge weights

**Fig 5:**
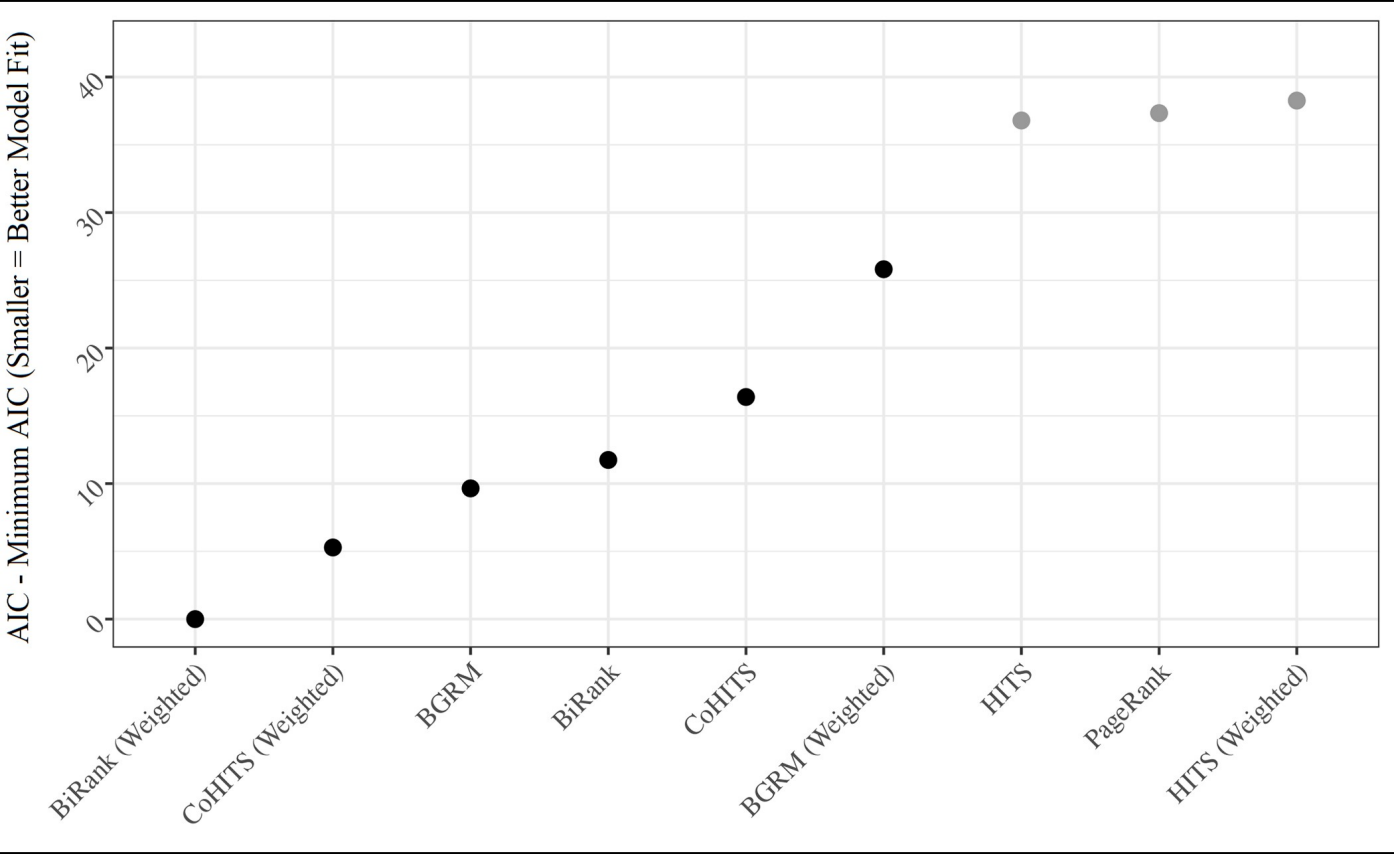
Model fit by rank parameter and MME-weighted edges. Dark points indicate parameter estimates that are statistically significant at p < 0.01. All parameter estimates have positive coefficients. Models control for age, gender (female), patient degree (number of providers), transitive ties, HEPC, HIV, cancer, psychological disorders, palliative care, and MAT use.

Finally, we demonstrate the effectiveness of incorporating meaningful edge weights into the bipartite centrality indices. We calculate the AIC values of our baseline models and models with centrality indices estimated on the MME-weighted network. The results in Fig 5 show that models with BiRank and CoHITS centrality indices on MME-weighted network resulted in lower AIC values than their unweighted counterparts. Even though MME-weights appear to substantially improve HITS’ raw association with opioid overdose (see Fig 4), the HITS model with MME-weights does not gain any improvement in fit, implying that MME-weights do not increase HITS’ ability to provide unique structural information about the network. Also, surprisingly, BGRM performs worse on the MME-weighted network than BGRM performed on the unweighted network.

To ensure that the improvement in centrality measures with MME-weighted edges is not simply a result of MME’s own direct association with the quantity of drugs consumed, we also estimate models that included MME as a covariate. These results are very similar to those displayed in Fig 5.

### Robustness analysis

To demonstrate that the findings above can generalize to different scenarios, we perform additional robustness analysis. We focus on the same geographical area but extract the data from 2012 quarter 3 to 2015 quarter 2. We run the same Cox proportional hazard models as before on this new dataset. The hazard ratios are reported in S2 Table; the model fit comparison is shown in S1 Fig. The results in them are qualitatively consistent with those in Table 1 and Fig 5, confirming the robustness of the findings.

## Discussion

In this study, we examine a patient-provider network defined by opioid prescriptions and compare how different centrality algorithms capture prescription drug-seeking behavior and predict subsequent opioid overdose. As shown in previous studies, network centrality better reflect the position of patients within the entire social structure of the patient-provider network than individual-level proxies of patient behavior, like degree and transitivity [17,21]. However, we argue that the extent to which any centrality index represents true prescription drug-seeking behavior is based on the extent to which the index reflects drug seekers’ behavior patterns. These patterns include the patients’ tendency to seek opioids from multiple providers simultaneously, to recommend and cluster around the same set of opioid providers, and to seek providers that prescribe higher quantities of potent opioids.

Although applying PageRank to the one-mode projected network is a common method for estimating node prominence in bipartite social networks and has been used previously for identifying opioid doctor shoppers [17,21], the comparison here reveals that some variants of a bipartite centrality index can better predict subsequent opioid overdose. Our analysis suggests that PageRank’s higher collinearity with the other model terms (in particular, transitive ties) makes it better at reflecting patients’ tendency to seek opioids from well-connected providers of opioid analgesics but worse at capturing patients’ preferences to seek opioids from many providers simultaneously. This shortcoming can be critical in the context of nonmedical prescription opioid use because a tendency to receive opioids from multiple providers simultaneously is the key characteristic of drug-seeking behavior according to the literature. In addition, it is non-trivial to incorporate the drug potency (MME) information into the PageRank estimates, limiting PageRank’s ability to capture the patients’ tendency to visit providers that prescribe high quantities of opioids.

By contrast, bipartite centrality indices CoHITS and BiRank yield better performance in predicting subsequent overdose events since they can better process the bipartite topology without introducing too much distortion and handle the outliers through proper normalization. Moreover, the bipartite centrality indices allow weighting patient edges by the potency of opioids (MME) and our analysis show that this enhancement further improves the performance of CoHITS and BiRank. This finding illustrates the advantages of weighting bipartite networks by relevant edge traits.

Not all bipartite centrality indices consistently perform better than PageRank in our analyses. For example, HITS performs worse than the other indices in predicting subsequent opioid overdose. As others suggested, the lack of a proper normalization scheme makes HITS susceptible to outliers [38,48], which may further lead to its weak performance. While the direct association between HITS and overdose appears to improve on the MME-weighted network (though the MME-weighted network has even more degree outliers), HITS’ increased association with opioid overdose is entirely mediated by other network controls, suggesting that HITS is still unable to pick up any distinctive structural information about the network. Put together, this suggests that despite its ability to reflect the bipartite topology of the patient-provider network, HITS is still a poor alternative to PageRank in this context.

BGRM shows mixed patterns: it predicts overdose better than PageRank and CoHITS with the unweighted patient-provider network in regression models while its direct association with opioid overdose is much weaker. Descriptive statistics and supplementary analyses suggest that BGRM’s advantage over CoHITS relies on the presence of controls for patient degree and transitive ties. We consider BGRM’s reliance on covariates to be a clear disadvantage in comparison to BiRank and CoHITS. More troubling, BGRM with MME-weighted edges perform worse than BGRM without MME-weights. This implies that BGRM’s normalization scheme prevents it from tracking onto patients’ preferences for providers of potent opioids. BGRM also has a negative correlation with transitive ties. Together, these patterns imply that BGRM tracks onto patients’ tendency to seek multiple providers simultaneously and to cluster around the same sets of opioid providers; however, BGRM does not appear to reflect preferences for high-quantity opioid providers.

Our analysis has some limitations. First, we do not know which patients truly engage in drug-seeking behavior. Instead, we use subsequent opioid overdose as a proxy. Although this methodology adheres with prior convention [17,21], it allows room for our estimates to be biased by network features that are related to opioid overdose but are not related to patient drug-seeking behavior. Second, it is unclear whether our estimates of prescription drug-seeking behavior are biased by missing information. Our medical claims database does not contain all patients within our region of analysis. However, centrality indices tend to be robust to missing information [63,64]. Third, our analysis does not explicitly account for other factors such as the geographical location of the patients and prescribers. Given that high-risk patients and prescribers tend to form geographic clusters, properly incorporating these factors into the framework could potentially improve the prediction power of the centrality measures.

When it comes to choosing the most appropriate centrality index, our finding may not generalize to other contexts. PageRank’s bias toward nodes with high-degree alters has some clear disadvantages for measuring drug-seeking in patient-provider networks, but it might not be problematic for scenarios where the structure of the network has a clearer hierarchy, a more core-periphery structure, or where node out-degree is not necessarily expected to be related to node prominence. Similarly, HITS and BGRM might be more suitable for other cases not considered here.

## Conclusion

This article compares various measures of node prominence in patient-provider networks to identify potential prescription drug-seeking behavior for nonmedical use. Our evidence suggests that some bipartite centrality indices may be excellent alternatives to PageRank in estimating node prominence in bipartite networks due to their capability of holistically capturing the bipartite topology and their flexibility to incorporate non-network traits to capture different aspects of the underlying social processes. Identifying individuals who are at risk of future opioid overdose is critical to population health in the U.S. We hope that these methods might be implemented to guide future prescription drug monitoring programs to reduce overdose mortality and improve access to addiction services.

Although we only provide one case study, we believe that bipartite centrality indices have demonstrated substantial advantages and will serve as useful tools for other insurance claims or electronic health records mining tasks such as identifying drug-seekers of other controlled substance and detecting insurance fraud and abuse [12,20,22,65] as well as for analyzing other bipartite networks such as power grids [66]. Our analysis shows that estimating centrality directly on bipartite networks offers theoretical advantages over applying traditional centrality measures like PageRank to one mode network projections. The variants of bipartite centrality index focus on different aspects of the network structure, allowing enough room for one to find an appropriate index for networks where node prominence is manifested differently. The analytic procedures and the explanation of how each centrality index works presented in this paper can aid the readers to experiment bipartite centrality indices in their studies. Finally, these indices are relatively easy to implement and deploy with modern statistical packages [62].

## Supporting information

S1 Fig Model fit by rank parameter and MME-weighted edges (2012 quarter 3 to 2015 quarter 2). (DOCX)

S1 Table Descriptive statistics of the variables used in the regression analysis. (DOCX)

S2 Table Cox proportional hazard models for opioid overdose (2012 quarter 3 to 2015 quarter 2). (DOCX)

## References

[1] Maxwell JC. The Prescription Drug Epidemic in the United States: A Perfect Storm. Drug and Alcohol Review. 2011;30(3):264–70. doi: 10.1111/j.1465-3362.2011.00291.x 21545556

[2] Peirce GL, Smith MJ, Abate MA, Halverson J. Doctor and Pharmacy Shopping for Controlled Substances. Medical Care. 2012;50:494–500. doi: 10.1097/MLR.0b013e31824ebd81 22410408

[3] Hedegaard H, Miniño AM, Warner M. Drug overdose deaths in the United States, 1999–2018. National Center for Health Statistics (U.S.), editor. 2020 Jan;(354). Available from: https://stacks.cdc.gov/view/cdc/84647. 32487285

[4] Compton WM, Boyle M, Wargo E. Prescription Opioid Abuse: Problems and Responses. Preventive Medicine. 2015;80:5–9. doi: 10.1016/j.ypmed.2015.04.003 25871819

[5] Han B, Compton WM, Jones CM, Cai R. Nonmedical Prescription Opioid Use and Use Disorders Among Adults Aged 18 Through 64 Years in the United States, 2003–2013. JAMA. 2015;314(14). doi: 10.1001/jama.2015.11859 26461997

[6] Martyres RF, Clode D, Burns JM. Seeking Drugs or Seeking Help? Escalating “Doctor Shopping” by Young Heroin Users before Fatal Overdose. Medical Journal of Australia. 2004;180(5):211–4. doi: 10.5694/j.1326-5377.2004.tb05887.x 14984339

[7] Yang Z, Wilsey B, Bohm M, Weyrich M, Roy K, Ritley D, et al. Defining Risk of Prescription Opioid Overdose: Pharmacy Shopping and Overlapping Prescriptions Among Long-Term Opioid Users in Medicaid. The Journal of Pain. 2015;16(5):445–53. doi: 10.1016/j.jpain.2015.01.475 25681095

[8] McDonald DC, Carlson KE. The ecology of prescription opioid abuse in the USA: geographic variation in patients’ use of multiple prescribers (“doctor shopping”). Pharmacoepidemiology and drug safety. 2014;23(12):1258–67. 25111716 10.1002/pds.3690 PMC4777341

[9] Nordmann S, Pradel V, Lapeyre-Mestre M, Frauger E, Pauly V, Thirion X, et al. Doctor shopping reveals geographical variations in opioid abuse. Pain Physician. 2013 Jan;16(1):89–100. 23340537

[10] Barnett ML, Olenski AR, Jena AB. Opioid-Prescribing Patterns of Emergency Physicians and Risk of Long-Term Use. New England Journal of Medicine. 2017;376(7):663–73. doi: 10.1056/NEJMsa1610524 28199807 PMC5428548

[11] McDonald DC, Carlson KE. Estimating the prevalence of opioid diversion by “doctor shoppers” in the United States. PLoS ONE. 2013;8(7):e69241. doi: 10.1371/journal.pone.0069241 23874923 PMC3714248

[12] Stein BD, Mendelsohn J, Gordon AJ, Dick AW, Burns RM, Sorbero M, et al. Opioid Analgesic and Benzodiazepine Prescribing among Medicaid-Enrollees with Opioid use Disorders: The Influence of Provider Communities. Journal of Addictive Diseases. 2017;36(1):14–22. doi: 10.1080/10550887.2016.1211784 27449904 PMC5366980

[13] Wilsey BL, Fishman SM, Gilson AM, Casamalhuapa C, Baxi H, Zhang H, et al. Profiling Multiple Provider Prescribing of Opioids, Benzodiazepines, Stimulants, and Anorexics. Drug and Alcohol Dependence. 2010;112(1–2):99–106. 20566252 10.1016/j.drugalcdep.2010.05.007

[14] Cepeda MS, Fife D, Chow W, Mastrogiovanni G, Henderson SC. Assessing Opioid Shopping Behaviour. Drug Safety. 2012;35(4):325–34. 22339505 10.2165/11596600-000000000-00000

[15] Pradel V, Frauger E, Thirion X, Ronfle E, Lapierre V, Masut A, et al. Impact of a Prescription Monitoring Program on Doctor-Shopping for High Dosage Buprenorphine. Pharmacoepidemiology and drug safety. 2009;18:36–43. doi: 10.1002/pds.1681 19040199

[16] Sansone RA, Sansone LA. Doctor Shopping: A Phenomenon of Many Themes. Innovations in Clinical Neuroscience. 2012;9(11–12). 23346518 PMC3552465

[17] Perry BL, Odabaş M, Yang K-C, Lee B, Kaminski P, Aronson B, et al. New means, new measures: assessing prescription drug-seeking indicators over 10 years of the opioid epidemic. Addiction [Internet]. 2021 [cited 2021 Oct 19];n/a(n/a). Available from: https://onlinelibrary.wiley.com/doi/abs/10.1111/add.15635 10.1111/add.15635 PMC8664959 34227707

[18] Morris BJ, Zumsteg JW, Archer KR, Cash B, Mir HR. Narcotic Use and Postoperative Doctor Shopping in the Orthopaedic Trauma Population. The Journal of Bone & Joint Surgery. 2014;96(15):1257–62. doi: 10.2106/JBJS.M.01114 25100772

[19] Thomas CP, Fullerton CA, Kim M, Montejano L, Lyman DR, Dougherty RH, et al. Medication-Assisted Treatment with Buprenorphine: Assessing the Evidence. Psychiatric Services. 2014;65(2):158–70. doi: 10.1176/appi.ps.201300256 24247147

[20] Ong M-S, Olson KL, Cami A, Liu C, Tian F, Selvam N, et al. Provider Patient-Sharing Networks and Multiple-Provider Prescribing of Benzodiazepines. Journal of General Internal Medicine. 2016;31(2):164–71. doi: 10.1007/s11606-015-3470-8 26187583 PMC4720655

[21] Perry BL, Yang KC, Kaminski P, Odabas M, Park J, Martel M, et al. Co-Prescription Network Reveals Social Dynamics of Opioid Doctor Shopping. PLoS ONE. 2019;14(10):e0223849. doi: 10.1371/journal.pone.0223849 31652266 PMC6814254

[22] Takahashi Y, Ishizaki T, Nakayama T, Kawachi I. Social Network Analysis of Duplicative Prescriptions: One-Month Analysis of Medical Facilities in Japan. Health Policy. 2016;120(3):334–41. doi: 10.1016/j.healthpol.2016.01.020 26876297

[23] Perry BL, Freeman P, Oser C, Martel M, Talbert J. Can Social Network Analysis be Used to Identify Doctor Shoppers? Value in Health. 2015;18(3).

[24] Wesson DR, Smith DE. Prescription Drug Abuse. Patient, Clinician, and Cultural Responsibilities. Western Journal of Medicine. 1990;152:613–6. 2349802 PMC1002420

[25] Borgatti SP, Everett MG. Network Analysis of 2-mode Data. Social Networks. 1997;19(3):243–69.

[26] Hanneman RA, Riddle M. Introduction to Social Network Methods. 2005.

[27] Moody J, Light R. A View from Above: The Evolving Sociological Landscape. The American Sociologist. 2006;37(2):67–86.

[28] Otte E, Rousseau R. Social Network Analysis: A Powerful Strategy, Also for the Information Sciences. Journal of Information Science. 2002;28(6):441–53.

[29] Wasserman S, Faust K, others. Social Network Analysis: Methods and Applications. Vol. 8. Cambridge University Press; 1994.

[30] Page L, Brin S, Motwani R, Winograd T. The PageRank Citation Ranking: Bringing Order to the Web. Stanford InfoLab. 1999.

[31] Bonacich P. Simultaneous Group and Individual Centralities. Social Networks. 1991;13(2):155–68.

[32] Latapy M, Magnien C, Vecchio ND. Basic Notions for the Analysis of Large Two-Mode Networks. Social Networks. 2008;30(1):31–48.

[33] Melamed D. Community Structures in Bipartite Networks: A Dual-Projection Approach. PLoS ONE. 2014;9(5):e97823. doi: 10.1371/journal.pone.0097823 24836376 PMC4023988

[34] Everett MG. Centrality and the Dual-Projection Approach for Two-Mode Social Network Data. Methodological Innovations. 2016;9(205979911663066).

[35] Zhou T, Ren J, Medo M, Zhang Y-C. Bipartite Network Projection and Personal Recommendation. Physical Review E. 2007;76(4). doi: 10.1103/PhysRevE.76.046115 17995068

[36] Lehmann S, Schwartz M, Hansen LK. Biclique communities. Physical review E. 2008;78(1):016108. doi: 10.1103/PhysRevE.78.016108 18764021

[37] Deng H, Lyu MR, King I. A Generalized Co-HITS Algorithm and Its Application to Bipartite Graphs. In: Proceedings of the 15th ACM SIGKDD International Conference on Knowledge Discovery and Data Mining. Paris, France: ACM Press; 2009. p. 239.

[38] He X, Gao M, Kan M-Y, Wang D. BiRank: Towards Ranking on Bipartite Graphs. IEEE Transactions on Knowledge and Data Engineering. 2017;29(1):57–71.

[39] Rui X, Li M, Li Z, Ma W-Y, Yu N. Bipartite Graph Reinforcement Model for Web Image Annotation. In: Proceedings of the 15th ACM International Conference on Multimedia. 2007. p. 585–94.

[40] Kleinberg JM. Authoritative Sources in a Hyperlinked Environment. Journal of the ACM. 1999;46(5):604–32.

[41] Friedkin NE, Johnsen EC. Two Steps to Obfuscation. Social Networks. 2014;39:12–3.

[42] Newman ME, Strogatz SH, Watts DJ. Random Graphs with Arbitrary Degree Distributions and their Applications. Physical Review E. 2001;64(2). doi: 10.1103/PhysRevE.64.026118 11497662

[43] Uzzi B, Spiro J. Collaboration and Creativity: The Small World Problem. American Journal of Sociology. 2005;111(2):447–504.

[44] Everett MG, Borgatti SP. The Dual-Projection Approach for Two-Mode Networks. Social Networks. 2013;35(2):204–10.

[45] Grassi R, Calderoni F, Bianchi M, Torriero A. Betweenness to Assess Leaders in Criminal Networks: New Evidence Using the Dual Projection Approach. Social Networks. 2019;56:23–32.

[46] Yildirim MA, Coscia M. Using random walks to generate associations between objects. PloS one. 2014;9(8):e104813. doi: 10.1371/journal.pone.0104813 25153830 PMC4143196

[47] Guillaume J-L, Latapy M. Bipartite Structure of all Complex Networks. Information Processing Letters. 2004;90(5):215–21.

[48] Devi P, Gupta A, Dixit A. Comparative Study of HITS and PageRank Link Based Ranking Algorithms. International Journal of Advanced Research in Computer and Communication Engineering. 2014;3(2):5749–54.

[49] Martin T, Zhang X, Newman ME. Localization and centrality in networks. Physical review E. 2014;90(5):052808. doi: 10.1103/PhysRevE.90.052808 25493835

[50] Compton WM, Jones CM, Baldwin GT. Relationship between Nonmedical Prescription-opioid use and heroin use. New England Journal of Medicine. 2016;374(2):154–63. doi: 10.1056/NEJMra1508490 26760086 PMC11784537

[51] Ali MM, Dowd WN, Classen T, Mutter R, Novak SP. Prescription Drug Monitoring Programs, Nonmedical Use of Prescription Drugs, and Heroin Use: Evidence from the National Survey of Drug Use and Health. Addictive Behaviors. 2017;69:65–77. doi: 10.1016/j.addbeh.2017.01.011 28152391

[52] Lilly JD. The Failure of Prescription Drug Monitoring Programs. Journal of American Physicians and Surgeons. 2019;24(2):56–9.

[53] Bahmani B, Chowdhury A, Goel A. Fast Incremental and Personalized PageRank. Proceedings of the VLDB Endowment. 2010;4(3):173–84.

[54] Boucher V, Fortin B. Some Challenges in the Empirics of the Effects of Networks. In: The Oxford Handbook on the Economics of Networks. 2016. p. 277–302.

[55] Fujimoto K, Chou C-P, Valente TW. The Network Autocorrelation Model Using Two-Mode Data: Affiliation Exposure and Potential Bias in the Autocorrelation Parameter. Social Networks. 2011;33(3):231–43. doi: 10.1016/j.socnet.2011.06.001 21909184 PMC3167212

[56] Mouw T, Entwisle B. Residential Segregation and Interracial Friendship in Schools. American Journal of Sociology. 2006;112(2):394–441.

[57] US Census Bureau. TIGER/Line Shapefiles [Internet]. Census.gov. [cited 2022 Feb 11]. Available from: https://www.census.gov/geographies/mapping-files/time-series/geo/tiger-line-file.html.

[58] Cox DR, Oakes D. Analysis of survival data. Vol. 21. CRC press; 1984.

[59] Allison PD. Event History Analysis: Regression for Longitudinal Event Data. Sage; 1984.

[60] Sakamoto Y, Ishiguro M, Kitagawa G. Akaike information criterion statistics. Dordrecht, The Netherlands: D Reidel. 1986;81(10.5555):26853.

[61] World Health Organization. International Classification of Diseases: Ninth Revision. 1978.

[62] Yang K-C, Aronson B, Ahn Y-Y. BiRank: Fast and Flexible Ranking on Bipartite Networks with R and Python. Journal of Open Source Software. 2020;5(51):2315. doi: 10.21105/joss.02315 34729449 PMC8559594

[63] Smith JA, Moody J. Structural Effects of Network Sampling Coverage I: Nodes Missing at Random. Social Networks. 2013;35(4):652–68. 10.1016/j.socnet.2013.09.003 PMC3846431 24311893

[64] Wang DJ, Shi X, McFarland DA, Leskovec J. Measurement Error in Network Data: A Re-classification. Social Networks. 2012;34(4):396–409.

[65] Gangopadhyay A, Chen S, Yesha Y. Detecting Healthcare Fraud through Patient Sharing Schemes. In: Dua S, Gangopadhyay A, Thulasiraman P, Straccia U, Shepherd M, Stein B, editors. Information Systems, Technology and Management. Berlin, Heidelberg: Springer; 2012. p. 421–6. (Communications in Computer and Information Science).

[66] Gurfinkel AJ, Silva DA, Rikvold PA. Centrality Fingerprints for Power Grid Network Growth Models. Physics Procedia. 2015 Jan 1;68:52–5.

