# Supplementary material for: Comparing measures of centrality in bipartite patient-prescriber networks: A study of drug seeking for opioid analgesics
Source: PLoS One. 2022 Aug 30;17(8):e0273569. doi: 10.1371/journal.pone.0273569 (PMC9426918; doi:10.1371/journal.pone.0273569)
Supplement: S1 Fig — (DOCX) [file pone.0273569.s001.docx]

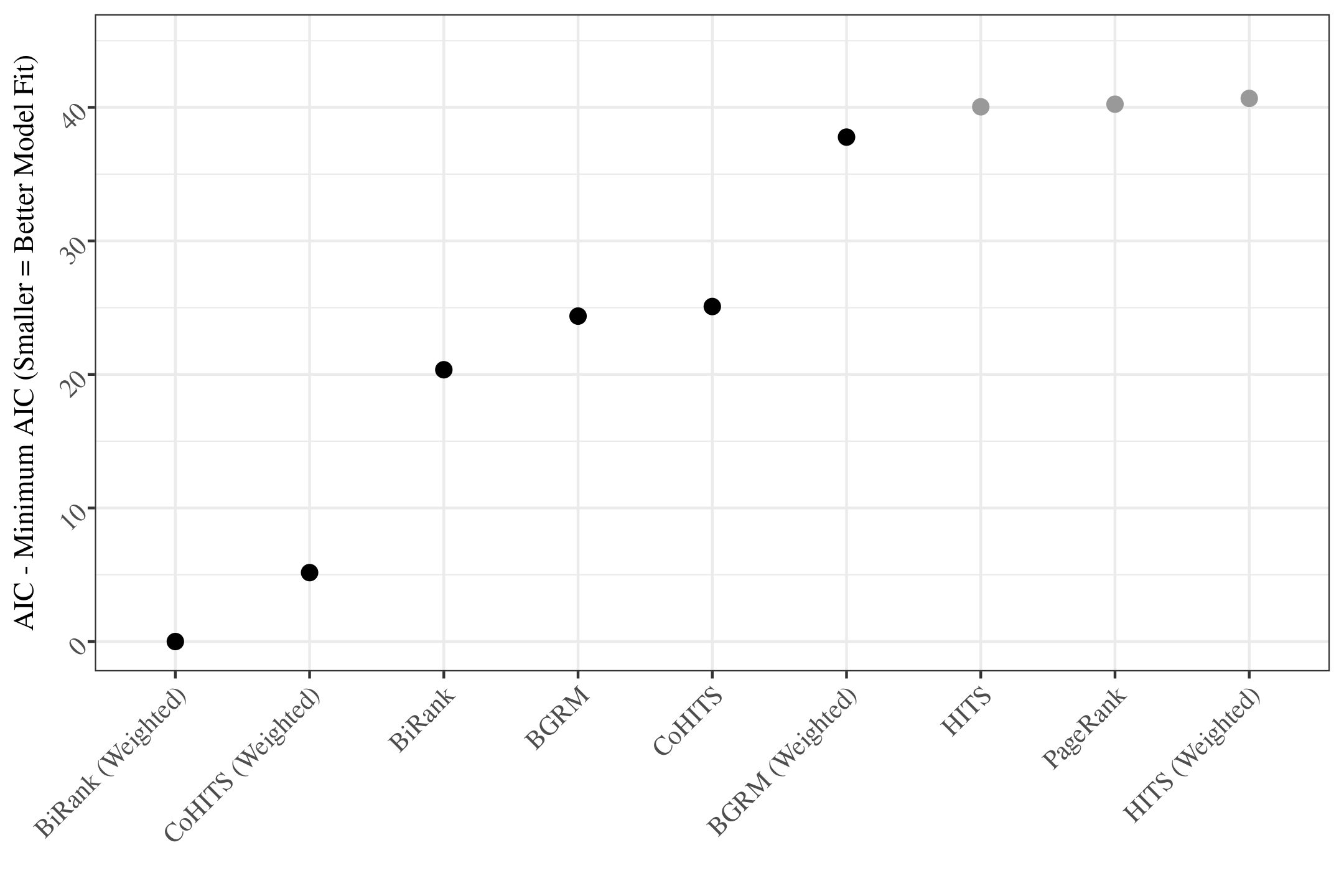


**S1 Fig. Model fit by rank parameter and MME-weighted edges (2012 quarter 3 to 2015 quarter 2).** Dark points indicate parameter estimates that are statistically significant at p < 0.01. All parameter estimates have positive coefficients. Models control for age, gender (female), patient degree (number of providers), transitive ties, HEPC, cancer, psychological disorders, palliative care, and MAT use.
