# Supplementary material for: Comparing measures of centrality in bipartite patient-prescriber networks: A study of drug seeking for opioid analgesics
Source: PLoS One. 2022 Aug 30;17(8):e0273569. doi: 10.1371/journal.pone.0273569 (PMC9426918; doi:10.1371/journal.pone.0273569)
Supplement: S1 Table — (DOCX) [file pone.0273569.s002.docx]

**S1 Table. Descriptive statistics of the variables used in the regression analysis.**

|  | **Mean** | **SD** | **Minimum** | **Maximum** |  |
| --- | --- | --- | --- | --- | --- |
| **Dependent Variable** |  |  |  |  |  |
| Opioid overdose | 0.00 | 0.01 | 0.00 | 1.00 |  |
| **Centrality Variables** |  |  |  |  |  |
| PageRank | 0.33 | 0.55 | 0.00 | 15.68 |  |
| HITS | 0.33 | 7.15 | 0.00 | 429.78 |  |
| CoHITS | 0.33 | 0.58 | 0.00 | 22.36 |  |
| BGRM | 0.33 | 0.50 | 0.00 | 2.57 |  |
| BiRank | 0.33 | 0.50 | 0.00 | 5.03 |  |
| **Control Variables** |  |  |  |  |  |
| Age | 50.13 | 20.53 | 0.00 | 89.00 |  |
| Female | 0.53 | 0.50 | 0.00 | 1.00 |  |
| Provider Count | 0.27 | 0.60 | 0.00 | 18.00 |  |
| Transitive Ties | 6.78 | 18.08 | 0.00 | 472.00 |  |
| Any Rx | 0.75 | 0.44 | 0.00 | 1.00 |  |
| HEPC | 0.00 | 0.03 | 0.00 | 1.00 |  |
| HIV | 0.00 | 0.03 | 0.00 | 1.00 |  |
| Cancer | 0.10 | 0.29 | 0.00 | 1.00 |  |
| Psych Disorder | 0.08 | 0.27 | 0.00 | 1.00 |  |
| Palliative Care | 0.00 | 0.03 | 0.00 | 1.00 |  |
| MAT User | 0.00 | 0.04 | 0.00 | 1.00 |  |
| Notes: Opioid overdose includes overdose through unclassified drugs. Centrality scores are normalized such that rank = rank/mean(rank). Transitive ties refer to patient-patient ties through providers. MAT user refers to individuals on medical assisted therapies. | | | | |  |
|  |  |  |  |  |  |
|  |  |  |  |  |  |
|  |  |  |  |  |  |
