# Supplementary material for: Comparing measures of centrality in bipartite patient-prescriber networks: A study of drug seeking for opioid analgesics
Source: PLoS One. 2022 Aug 30;17(8):e0273569. doi: 10.1371/journal.pone.0273569 (PMC9426918; doi:10.1371/journal.pone.0273569)
Supplement: S2 Table — (DOCX) [file pone.0273569.s003.docx]

**S2 Table. Cox proportional hazard models for opioid overdose (2012 quarter 3 to 2015 quarter 2).**

|  | **Baseline** | **PageRank** | **HITS** | **CoHITS** | **BGRM** | **BiRank** |
| --- | --- | --- | --- | --- | --- | --- |
| **Centrality** | - | 1.058 | 0.958 | 1.156*** | 1.223*** | 1.204*** |
| **Demographics** |  |  |  |  |  |  |
| Age | 0.841** | 0.847** | 0.838** | 0.860** | 0.849** | 0.862** |
| Female | 0.880 | 0.878 | 0.881 | 0.879 | 0.879 | 0.878 |
| **Network Proxies** |  |  |  |  |  |  |
| Degree (# Providers) | 1.710*** | 1.682*** | 1.712*** | 1.513*** | 1.584*** | 1.482*** |
| Transitive Ties^a^ | 1.460*** | 1.416*** | 1.474*** | 1.522*** | 1.643*** | 1.562*** |
| **Related Disorders** |  |  |  |  |  |  |
| HEPC | 1.589 | 1.556 | 1.575 | 1.412 | 1.538 | 1.416 |
| Cancer | 0.980 | 0.980 | 0.981 | 0.959 | 0.957 | 0.953 |
| Psych Disorder | 5.234*** | 5.221*** | 5.242*** | 5.164*** | 5.199*** | 5.152*** |
| Palliative Care | 3.841*** | 3.860*** | 3.779*** | 3.541*** | 3.544*** | 3.487*** |
| MAT User^b^ | 4.739*** | 4.786*** | 4.777*** | 4.659*** | 4.581*** | 4.612*** |
| AIC | 13655 | 13655 | 13655 | 13640 | 13639 | 13635 |

* = p < 0.05, ** = p < 0.01, *** = p < 0.001. Time-to-event is based on one quarter intervals between 2012 quarter 3 and 2015 quarter 2. There are n = 1,749,600 patient-quarters. The number of overdose events is 560. The parameters are standardized and reported as hazard ratios. The binary variable “HIV” is removed from these models because patients with HIV and patient with opioid overdose do not overlap in this time period. a) Number of patient-patient ties through providers. b) Patient is receiving medication assisted therapy for opioid use disorder.
